# Machine learning-based ultrasound radiomics for predicting *TP53* mutation status in hepatocellular carcinoma

**DOI:** 10.3389/fmed.2025.1565618

**Published:** 2025-04-28

**Authors:** Didi Bu, Shaobo Duan, Shanshan Ren, Yujing Ma, Yuanyuan Liu, Yahong Li, Xiguo Cai, Lianzhong Zhang

**Affiliations:** ^1^Department of Ultrasound, Zhengzhou University People’s Hospital, Henan Provincial People’s Hospital, Zhengzhou University, Zhengzhou, China; ^2^Department of Health Management, Henan Provincial People’s Hospital, Zhengzhou, China; ^3^Department of Ultrasound, Henan Provincial People’s Hospital, Zhengzhou, China; ^4^Department of Ultrasound, Henan University People’s Hospital, Henan Provincial People’s Hospital, Zhengzhou, China; ^5^Henan Rehabilitation Clinical Medical Research Center, Henan Provincial People’s Hospital, Zhengzhou, China; ^6^Henan Key Laboratory for Ultrasound Molecular Imaging and Artificial Intelligence Medicine, Henan Provincial People’s Hospital, Zhengzhou, China

**Keywords:** radiomics, hepatocellular carcinoma, machine learning, *TP53*, ultrasonography

## Abstract

**Objectives:**

To explore the utility of machine learning-based ultrasound radiomics for predicting *TP53* gene mutation in hepatocellular carcinoma (HCC).

**Methods:**

154 HCC patients with 182 lesions from 2019 to 2024 were reviewed retrospectively. All lesions were randomly split into the training set (*n* = 129) and the test set (*n* = 53), and ultrasound radiomics features were extracted and selected. Extreme gradient boosting tree (XGBoost), decision tree (DT), random forest (RF), support vector machine (SVM), and logistic regression (LR) were used to construct the ultrasound radiomics models, the clinical models, and the combined models. The predictive performance of various models was evaluated by the area under the curve (AUC), accuracy, calibration curve, and decision curve analysis (DCA).

**Results:**

Among the 182 lesions, 102 were confirmed as mutant *TP53* and 80 were confirmed as wild-type *TP53*. The ultrasound radiomics model obtained an AUC of 0.778 and an accuracy of 0.774 in the test set. The clinical model achieved an AUC of 0.761 and an accuracy of 0.710 in the test set. Notably, integrating clinical features with ultrasound radiomics further enhanced predictive performance. The XGBoost-based combined model exhibited the highest predictive performance among all models, achieving an AUC of 0.846 and an accuracy of 0.823 in the test set. The decision curve analysis and calibration curve revealed that the XGBoost-based combined model provided the highest clinical benefit and exhibited strong predictive consistency.

**Conclusion:**

Machine learning-based ultrasound radiomics signatures accurately predict *TP53* gene mutations in HCC. The XGBoost-based combined model, which combined ultrasound radiomics features with clinical features, showed the best performance and represented a promising noninvasive approach for screening *TP53*-mutated HCC.

## 1 Introduction

Hepatocellular carcinoma (HCC) ranks third among cancer-related causes of mortality worldwide, posing a significant public health challenge (1). Surgical resection and liver transplantation remain the primary treatment options for HCC. However, the postoperative recurrence rate reaches up to 70% within 5 years (2). Despite advancements in immunotherapy that have expanded treatment options for HCC, the prognosis remains poor, with a five-year survival rate of only 15% (3). The poor prognosis of HCC is largely attributable to the highly heterogeneous and aggressive biological behavior of the tumor (4). Numerous studies have identified various tumor-specific gene mutations in HCC, which play a crucial role in regulating its biological behavior (5–7). Characterizing the genetic profile of HCC offers valuable insights for developing personalized treatment strategies and assessing prognosis.

*TP53* is a crucial tumor suppressor gene that regulates multiple signaling pathways and plays a key role in cellular processes, including apoptosis, cellular senescence, and DNA repair (8, 9). *TP53* mutations are the most prevalent genetic alterations in HCC, occurring in 15–40% of advanced cases (10). Mutations in *TP53* result in the loss of its regulatory function, thereby promoting tumorigenesis (11). Moreover, *TP53* mutations cause excessive nuclear accumulation of the p53 protein, which serves as a specific indicator of malignancy (12). *TP53* mutations are associated with HCC tumor staging, elevated AFP levels, poor prognosis, and vascular invasion (13–16). Mutant *TP53* enhances the aggressiveness and metastatic potential of HCC by inducing epithelial-mesenchymal transition (EMT) (17). Recent studies have demonstrated that *TP53* status influences the tumor immune microenvironment (TIME) (18–21). Specifically, wild-type *TP53* fosters a tumor-suppressive microenvironment, whereas mutant *TP53* contributes to an immunosuppressive microenvironment and promotes tumor immune evasion (22). Furthermore, *TP53* has emerged as a promising target for antitumor therapies, demonstrating significant clinical potential in HCC-targeted treatments (23). Thus, identifying *TP53* mutation status is crucial for guiding personalized treatment strategies and improving patient prognosis. Specifically, it enables clinicians to tailor therapeutic approaches, including surgical planning, adjuvant therapy selection, and follow-up monitoring, thereby optimizing clinical outcomes for HCC patients with different *TP53* statuses.

In clinical practice, preoperative assessment of *TP53* gene status primarily relies on immunohistochemistry (IHC) of biopsy tissue (24). However, spatial sampling errors during biopsy procedures may limit the accuracy of detection results, failing to fully capture tumor heterogeneity (25). Moreover, invasive procedures pose risks of bleeding, infection, and potential tumor dissemination (26). Therefore, there is an urgent need for a non-invasive approach capable of accurately predicting *TP53* gene status in HCC prior to surgery.

As an emerging technology, radiomics enables the high-throughput extraction of quantitative features from medical images, which provides more information on tumor phenotypes in a noninvasive manner from a multi-dimensional and multi-space perspective (27, 28). Radiomics overcomes the limitations of traditional molecular detection techniques and enables the comprehensive evaluation of the biological characteristics of tumors in a non-invasive manner (29). Previous studies have demonstrated the favorable performance of radiomics models based on computed tomography (CT) and magnetic resonance imaging (MRI) images in predicting the *TP53* gene status in rectal cancer and endometrial cancer (30, 31). However, limited radiomics studies have focused on predicting *TP53* gene status in HCC. To date, only one study has highlighted the potential of CT-derived texture features in predicting *TP53*-mutated HCC (32). Ultrasound is a radiation-free real-time imaging technology with convenient operation and low economic cost, making it highly valuable for early screening and prognostic monitoring of HCC (33, 34). The integration of ultrasound and radiomics has shown significant potential in the diagnosis and treatment monitoring of HCC. It has been widely applied in pathological grading, therapeutic efficacy assessment, and biomarker prediction in HCC (35–37). However, the ultrasound radiomics features of *TP53*-mutated HCC have not been investigated.

The study aims to investigate the potential of ultrasound radiomics models to predict *TP53* mutation status, in the hope of offering a novel method to promote the precision diagnosis and treatment of HCC.

## 2 Methods

### 2.1 Case inclusion

The ethics committee of our hospital granted approval for this retrospective study [Ethical number: (2021) Ethics Application No. (01)], and informed consent was waived.

**Figure 1:**
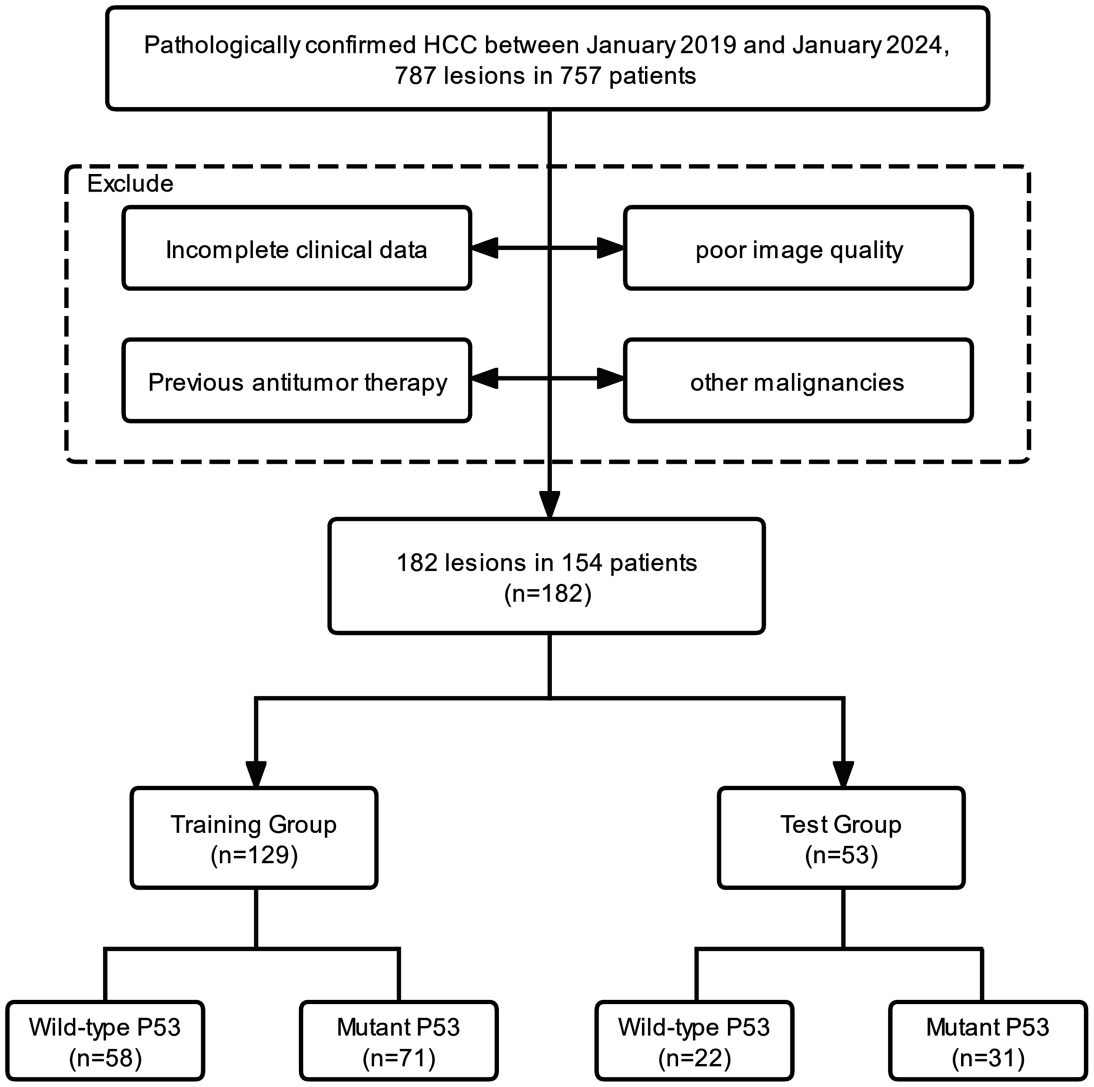
Flowchart of study inclusion and exclusion.

In this study, we consecutively evaluated patients who were postoperative histopathologically confirmed as HCC from January 2019 to January 2024 in our hospital. The inclusion criteria were as follows: (1) patients with pathologically confirmed HCC; (2) IHC results include p53; (3) liver ultrasound within 1 month before surgery; (4) no history of previous antitumor treatment; and (5) no history of other malignancies. The inclusion and exclusion process of the study is illustrated in Figure 1. Finally, 182 lesions from 154 patients were enrolled in this study. All lesions were partitioned into a training set (*n* = 129) and a test set (*n* = 53) by stratified sampling at a ratio of 7:3. The training set was used to train the model, and the test set was used to verify the model performance.

### 2.2 Clinical data and IHC

Clinical data of patients were collected from the electronic health record management system, including demographic characteristics such as age, sex, and preoperative laboratory parameters such as hepatitis B surface antigen (HBsAg), alpha-fetoprotein (AFP), aspartate aminotransferase (AST), alanine aminotransferase (ALT), total bilirubin (TBIL), albumin level (ALB), and prothrombin time (PT), as well as conventional ultrasound features such as lesion echo signal, lesion diameter, and doppler flow signal.

*TP53* gene status was evaluated based on p53 IHC expression patterns. Two pathologists independently assessed p53 expression following a previously described method (16). Both pathologists were blinded to the patient’s clinical and imaging data. p53 expression was evaluated according to the proportion of tumor cells with positive nuclear staining. Positive expression was defined as 10% or more of tumor cells with positive nuclear staining. Abnormal complete deletion was defined as the complete absence of tumor cell staining, with positive staining in internal controls (normal stromal cells). Negative expression was defined as less than 10% of tumor cells with positive nuclear staining. Positive expression or abnormal complete deletion were identified as mutant *TP53*, while negative expression was identified as wild-type *TP53* (24).

The preparation of p53 immunohistochemical sections and the detection methods for experimental indicators are detailed in Supplementary material 1.

### 2.3 Image acquisition

The ultrasound examination was performed following a standardized protocol. The ultrasound examination was conducted using Philips EPIQ 7, GE Vivid E9, or HIVISION Ascendus (C715; frequency range: 1–5 MHz). All patients fasted for 8 h and underwent liver ultrasound in the supine position. The maximum diameter, echo signal, and Doppler flow signal of lesions were assessed by two radiologists with 5 years of liver ultrasound experience. Both radiologists were blinded to the clinical and pathological information of the patients. The image indicating the maximum diameter of the lesion was saved in digital imaging and communications in medicine (DICOM) format for subsequent image segmentation. In total, 182 ultrasound images from 182 lesions were included for further analysis.

### 2.4 Image segmentation

**Figure 2:**
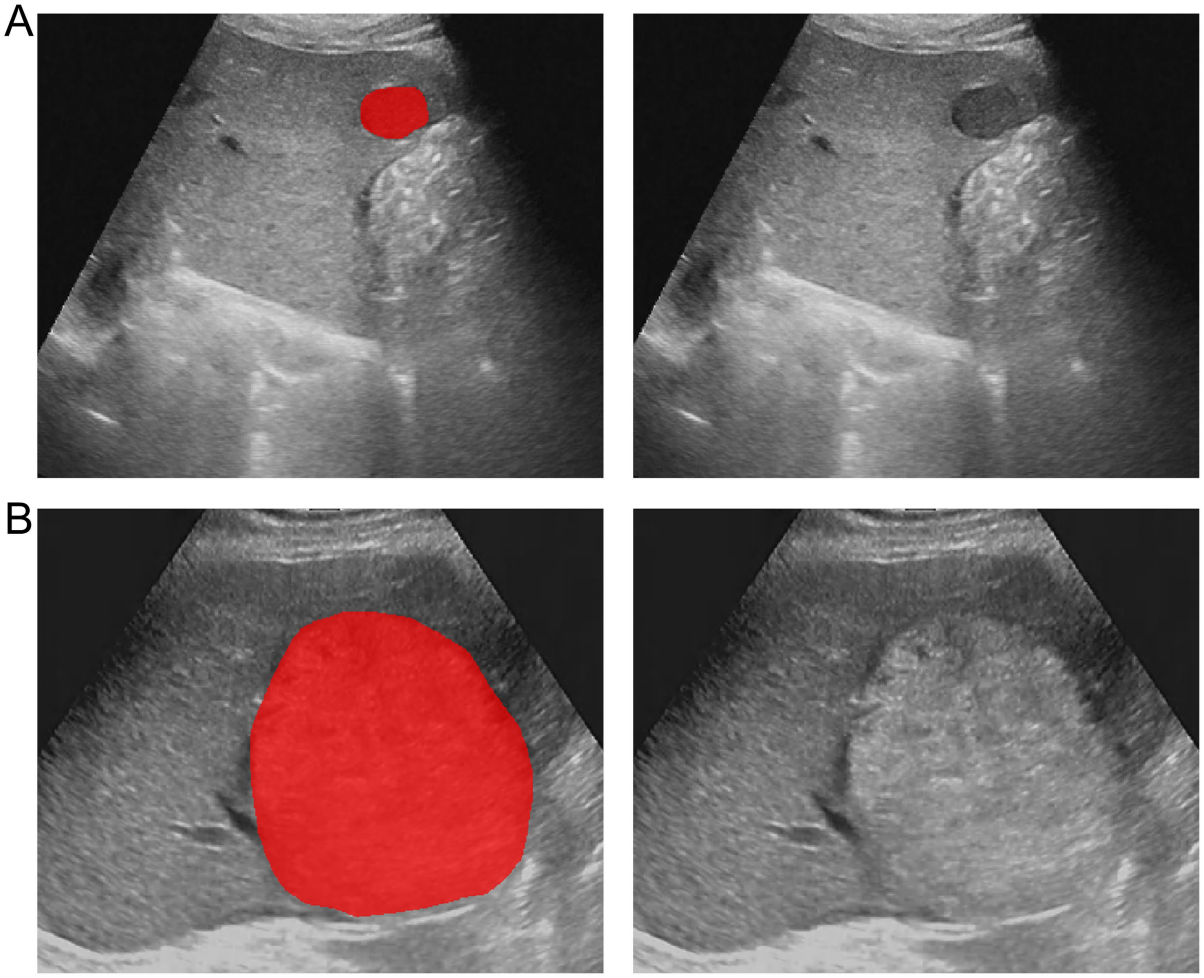
Example of delineating region of interest (ROI) on grayscale ultrasound images. **(A)** The lesion segmentation image and original image of a patient with wild-type *TP53*. **(B)** The lesion segmentation image and original image of a patient with mutant *TP53*.

The region of interest (ROI) of the HCC lesions was manually segmented using ITK-SNAP software (version 3.8.0) (38). The ROIs of all lesions were manually delineated by a radiologist along the tumor margin. To assess the reproducibility of the features, 30 HCC lesions were randomly selected and their ROIs were independently delineated by another radiologist. Both radiologists were blinded to the clinical and pathological information of the patients. The intraclass correlation coefficient (ICC) was calculated to assess the reproducibility of the features. An ICC value closer to 1 indicates higher reliability. Only features with an ICC value of ≥ 0.80 were included in the subsequent feature selection. Figure 2 illustrates the representative segmentation results of the HCC lesions.

### 2.5 Image preprocessing

Before extracting features, the images were preprocessed to ensure isotropy. First, the ultrasound images were normalized using the mean and standard deviation to mitigate the effects of variations in scanners and parameters. Subsequently, the images were resampled using the B-spline interpolation algorithm, standardizing the pixel size of all images to 1 mm × 1 mm. This step ensured uniform spatial resolution across all images. Finally, the images were subjected to gray-level discretization to constrain the gray-level value of each pixel in the interval of [0, 255] (39).

### 2.6 Feature extraction and selection

The original images were processed with 14 types of filters for noise reduction, and derived images were obtained. Ultrasound radiomics features were subsequently extracted from both the original and derived images utilizing the Pyradiomics package (version 2.1.2) in Python. The features were categorized into the following 7 classes: (1) first-order features, (2) shape features, (3) gray-level dependence matrix (GLDM), (4) gray-level co-occurrence matrix (GLCM), (5) gray-level run length matrix (GLRLM), (6) gray-level size-zone matrix (GLSZM), and (7) neighboring gray-tone-difference matrix (NGTDM). Except for the shape features, all other ultrasound radiomics features were computed from the original and derived images. To ensure uniform data distribution, the feature data were subjected to Z-score normalization.

Since the extracted features were high-dimensional, this may result in computational inefficiency and overfitting. Therefore, feature selection was required to screen the features most relevant to the model performance. First, the features with an ICC value of less than 0.8 were removed. Second, the variance threshold method was employed to exclude features with zero variance. Then, the maximal information coefficient (MIC) was calculated to evaluate the correlation between features and the target variables, and features with an MIC value of zero were eliminated. Finally, 24 features with the highest information were screened using the embedded method in combination with extreme gradient boosting (XGBoost).

### 2.7 Model establishment and evaluation

To address the imbalance in the dataset, we employed synthetic oversampling techniques (SMOTE) (40). The Python scikit-learn package (version 0.23.2) was utilized to build the model. Since each algorithm has distinct core principles and areas of applicability, their performance varies across different types of data. Five supervised learning classifiers were employed to build the ultrasound radiomics models, clinical models, and combined models to identify the optimal model, namely extreme gradient boosting (XGBoost), decision tree (DT), random forest (RF), support vector machine (SVM), and logistic regression (LR). Hyperparameter tuning is crucial for optimizing the performance of machine learning models. We applied random search and grid search methods to select the optimal hyperparameters for the machine learning models (Supplementary material 2).

First, the ultrasound radiomics models were developed using the 24 screened radiomics features. Then, clinical models were constructed using the clinical features of patients, such as sex, age, HBsAg, Child-Pugh class, AFP, AST, ALT, ALB, GGT, PT, liver cirrhosis, splenomegaly, tumor diameter. Finally, the combined models were developed by incorporating clinical characteristics and radiomics features.

The predictive performance of each model was analyzed using the area under the curve (AUC) and accuracy. Decision curve analysis (DCA) was used to calculate the clinical net benefits of the optimal ultrasound radiomics model, the optimal clinical model, and the optimal combined model, thereby evaluating the clinical value of all three models. The calibration curve was employed to assess the calibration degree of the optimal model.

**Figure 3:**
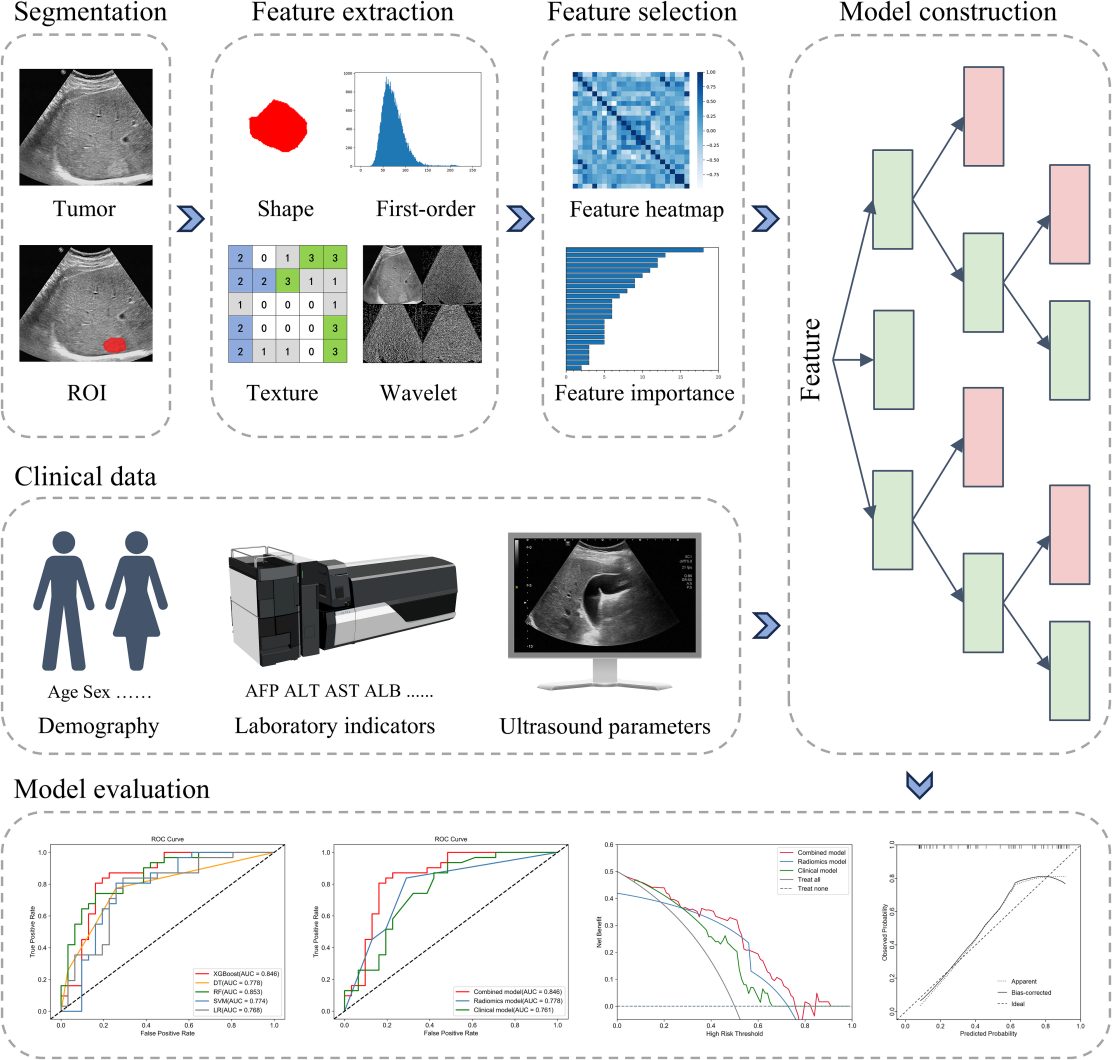
The radiomics workflow. ROI, region of interest; AFP, alpha-fetoprotein; ALT, alanine aminotransferase; AST, aspartate aminotransferase; ALB, albumin; AUC, the area under the curve.

The study procedure is illustrated in Figure 3.

### 2.8 Statistical analysis

SPSS 26.0 and R 4.4.1 were employed for statistical analysis. Continuous variables were analyzed using the t-test or Mann–Whitney U test. Categorical variables were analyzed using the Chi-square test. Statistical significance was defined as *p* < 0.05.

## 3 Results

### 3.1 Clinical characteristics of lesions

In this study, a total of 182 lesions were finally included, with 102 classified as mutant *TP53* and 80 as wild-type *TP53*. The training set and test set, respectively, comprised 129 and 53 lesions. The clinical characteristics of wild-type *TP53* and mutant *TP53* in the training and test sets are presented in Table 1. In the training set, AST existed a significant difference in the two groups (*p* < 0.05). In the test set, significant differences were observed in AFP levels and tumor diameter between the two groups (*p* < 0.05). In addition, we conducted a comparison of the clinical features between the training set and the test set. Except for portal hypertension and TBIL, there were no notable differences in other clinical and conventional ultrasound features between the training and test sets (Supplementary Table 1).

**Table 1:** Clinical characteristics of *TP53* status in the training set and the test set.

Characteristic	Training set (*n* = 129)			Test set (*n* = 53)		
	Wt *TP53* (*n* = 58)	Mut *TP53* (*n* = 71)	*p*-value	Wt *TP53* (*n* = 22)	Mut *TP53* (*n* = 31)	*p*-value
Age (years)	59.55 ± 10.18	57.11 ± 8.60	0.143	58.09 ± 11.04	56.32 ± 7.15	0.514
Sex			0.172			0.720
Male	49(84.50)	53(74.60)		19(86.40)	25(80.60)	
Female	9(15.50)	18(25.40)		3(13.60)	6(19.40)	
HBsAg			0.802			0.025
Negative	15(25.90)	17(23.90)		7(31.80)	2(6.50)	
Positive	43(74.10)	54(76.10)		15(68.20)	29(93.50)	
Child-Pugh class			0.213			0.686
A	49(84.50)	65(91.50)		20(90.90)	26(83.90)	
B-C	9(15.50)	6(8.50)		2(9.10)	5(16.10)	
Liver cirrhosis			0.681			0.120
No	6(10.30)	9(12.70)		1(4.50)	7(22.60)	
Yes	52(89.70)	62(87.30)		21(95.50)	24(77.40)	
Portal hypertension			0.878			0.724
No	36(62.10)	45(63.40)		17(77.30)	26(83.90)	
Yes	22(37.80)	26(36.60)		5(22.70)	5(16.10)	
AFP (ng/mL)			0.173			0.027
<400	44(75.90)	46(64.80)		19(86.40)	18(58.10)	
>400	14(24.10)	25(35.20)		3(13.60)	13(41.90)	
ALT (U/L)	27.85(22.95,39.20)	27.70(21.30,55.90)	0.581	30.00(19.95,51.08)	30.80(22.60,50.40)	1.000
AST (U/L)	30.15(22.68,46.00)	38.20(24.30,59.30)	0.044	30.55(21.63,49.65)	32.10(24.20,44.30)	0.396
ALB (g/L)	39.26 ± 5.43	39.33 ± 5.36	0.943	39.04 ± 6.02	38.65 ± 6.70	0.831
TBIL (μmol/L)	13.60(10.43,19.88)	13.80(9.40,19.60)	0.541	11.50(7.45,16.88)	11.40(8.90,13.80)	0.921
GGT (U/L)	57.65(33.28,93.30)	60.10(24.50,142.60)	1.000	73.05(33.93,135.48)	58.30(29.00,111.60)	0.613
PT (s)	12.65(11.98,13.93)	12.40(11.80,13.20)	0.233	12.40(11.98,12.80)	12.40(11.60,13.30)	0.906
Splenomegaly			0.701			0.454
No	33(56.90)	38(53.50)		15(68.20)	18(58.10)	
Yes	25(43.10)	33(46.50)		7(31.80)	13(41.90)	
Tumor diameter (mm)	34.00(24.00,53.00)	34.00(19.00,64.00)	0.755	30.50(18.75,40.25)	40.00(24.00,65.00)	0.034
Echo signal			0.929			0.301
Low	33(56.90)	38(53.50)		10(45.50)	10(32.30)	
Equal	9(15.50)	12(16.90)		6(27.30)	6(19.40)	
High	16(27.60)	21(29.60)		6(27.30)	15(48.40)	
Margin			0.677			0.089
Clear	38(65.50)	44(62.00)		9(40.90)	20(64.50)	
Obscure	20(34.50)	27(38.00)		13(59.10)	11(35.50)	
Shape			0.217			0.908
Regular	30(51.70)	29(40.80)		11(50.00)	15(48.40)	
Irregular	28(48.30)	42(59.20)		11(50.00)	16(51.60)	
Doppler flow signal			0.802			0.454
No	38(65.50)	48(67.60)		15(68.20)	18(58.10)	
Yes	20(34.50)	23(32.40)		7(31.80)	13(41.90)	
Tumor location			0.602			0.445
Left lobe	11(19.00)	11(15.50)		2(9.10)	6(19.40)	
Right lobe	47(81.00)	60(84.50)		20(90.90)	25(80.60)	

Wt TP53, wild-type TP53; Mut TP53, mutant TP53; HBsAg, hepatitis B surface antigen; AFP, alpha-fetoprotein; ALT, alanine aminotransferase; AST, aspartate aminotransferase; ALB, albumin; TBIL, total bilirubin; GGT, Gamma-glutamyltransferase; PT, prothrombin time.

### 3.2 Feature extraction and selection

**Figure 4:**
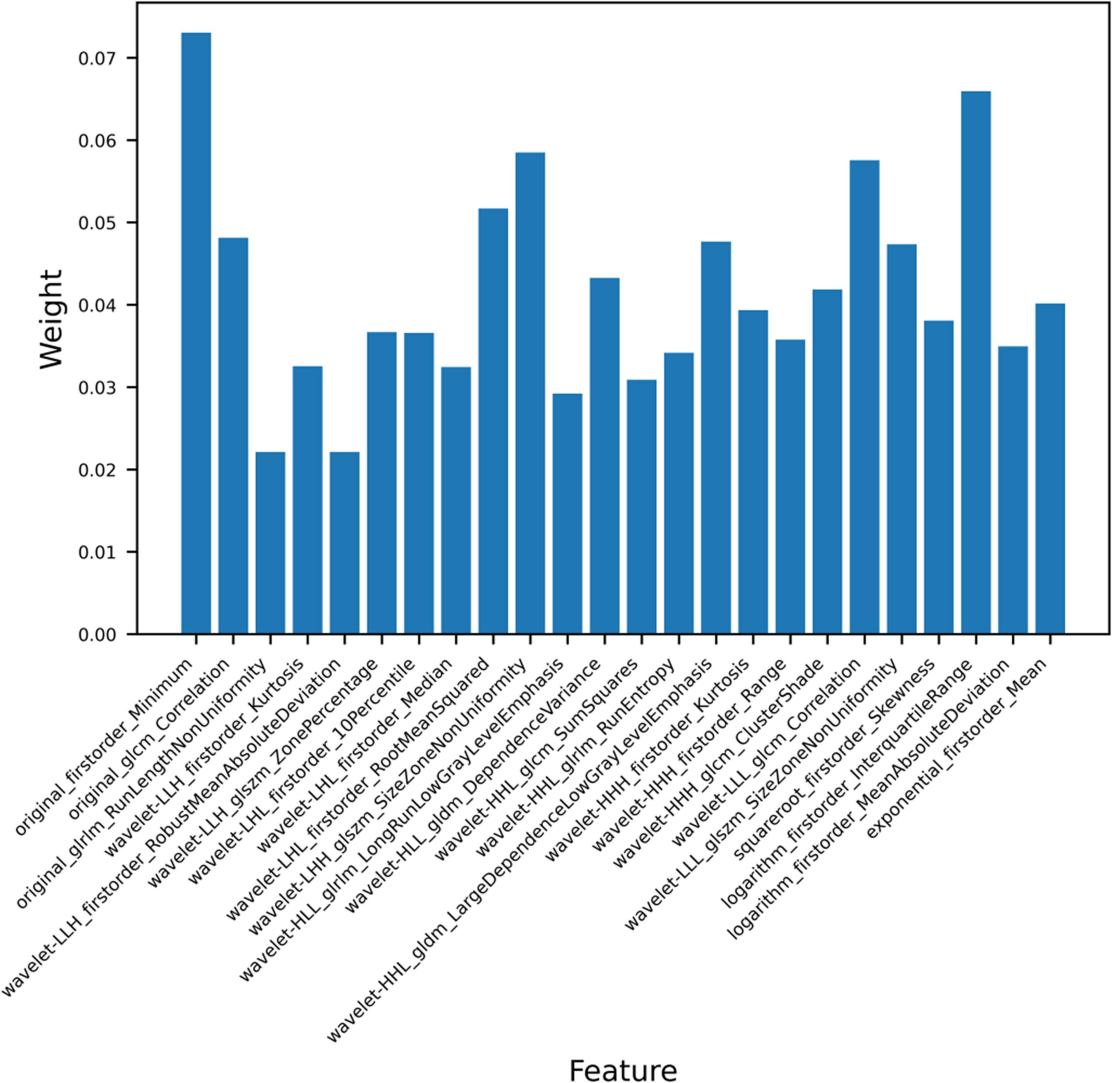
Weight histogram of 24 the radiomics features.

We extracted a total of 1,409 ultrasound radiomics features from the images. Of the extracted features, 256 features were eliminated due to ICC < 0.8. Next, 20 features exhibiting zero variance and 612 features with MIC value of zero were removed by applying the variance threshold and mutual information method. After further dimensionality reduction using the embedded method and XGBoost, the 24 most relevant radiomics features were finally selected (Figure 4). The detailed characteristics are provided in Supplementary Table 2.

### 3.3 Performance of ultrasound radiomics models and clinical models

**Figure 5:**
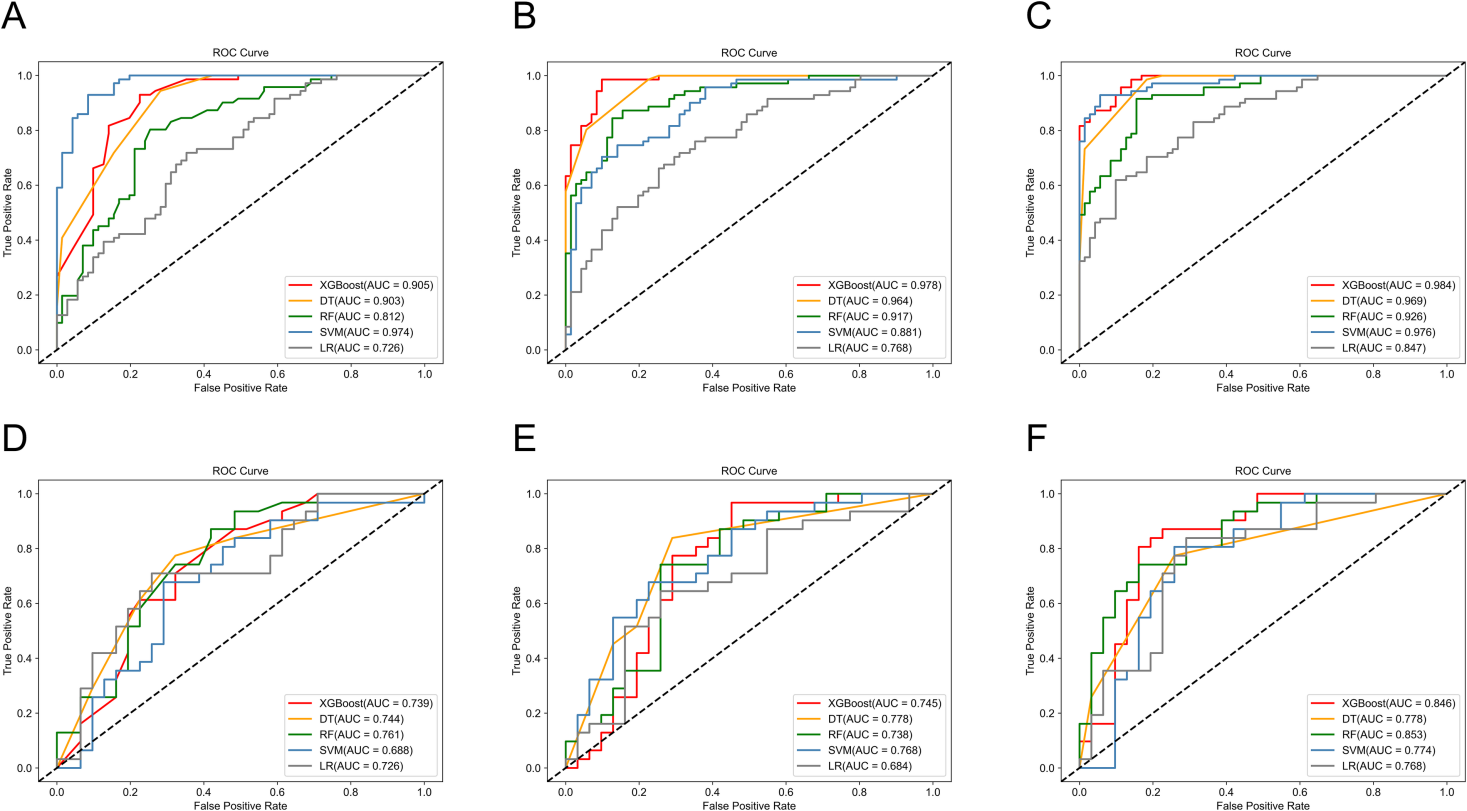
The ROC curves of machine learning models in the training set of **(A)** the clinical models, **(B)** the radiomics models, and **(C)** the combined models, and the test set of **(D)** the clinical models, **(E)** the radiomics models, and **(F)** the combined models.

We used five machine learning algorithms (XGBoost, DT, RF, SVM, and LR) to build ultrasound radiomics models and clinical models, and analyze and compare their predictive performance. Table 2 provides the detailed predictive performance of the ultrasound radiomics models and the clinical models. The ROC curves of the models are illustrated in Figures 5A,B,D,E.

**Table 2:** The performance of the models in the training set and the test set.

Model		Training set					Test set				
Group	Classifier	ACC	SEN	SPE	AUC (95%CI)	*p*-value	ACC	SEN	SPE	AUC (95%CI)	*p*-value
Clinical	XGBoost	0.845	0.915	0.775	0.905 (0.844–0.948)	<0.0001	0.661	0.645	0.677	0.739 (0.612–0.842)	0.0002
	DT	0.831	0.944	0.718	0.903 (0.842–0.946)	<0.0001	0.726	0.774	0.677	0.744 (0.617–0.846)	0.0001
	RF	0.761	0.803	0.718	0.812 (0.738–0.872)	<0.0001	0.710	0.742	0.677	0.761 (0.635–0.860)	<0.0001
	SVM	0.894	0.944	0.845	0.974 (0.933–0.994)	<0.0001	0.661	0.710	0.613	0.688 (0.557–0.800)	0.0071
	LR	0.669	0.718	0.620	0.726 (0.645–0.798)	<0.0001	0.661	0.710	0.613	0.726 (0.598–0.832)	0.0006
Radiomics	XGBoost	0.930	0.958	0.901	0.978 (0.938–0.995)	<0.0001	0.710	0.710	0.710	0.745 (0.618–0.847)	0.0003
	DT	0.880	0.986	0.775	0.964 (0.919–0.988)	<0.0001	0.774	0.839	0.710	0.778 (0.655–0.874)	<0.0001
	RF	0.838	0.873	0.803	0.917 (0.859–0.957)	<0.0001	0.742	0.742	0.742	0.738 (0.611–0.841)	0.0003
	SVM	0.775	0.746	0.803	0.881 (0.816–0.929)	<0.0001	0.726	0.677	0.774	0.768 (0.643–0.866)	<0.0001
	LR	0.704	0.704	0.704	0.768 (0.690–0.835)	<0.0001	0.661	0.645	0.677	0.684 (0.553–0.796)	0.0084
Combined	XGBoost	0.923	0.958	0.887	0.984 (0.946–0.997)	<0.0001	0.823	0.806	0.839	0.846 (0.732–0.925)	<0.0001
	DT	0.901	0.986	0.817	0.969 (0.926–0.991)	<0.0001	0.758	0.774	0.742	0.778 (0.655–0.874)	<0.0001
	RF	0.866	0.915	0.817	0.926 (0.870–0.963)	<0.0001	0.774	0.710	0.839	0.853 (0.740–0.930)	<0.0001
	SVM	0.887	0.944	0.831	0.976 (0.936–0.994)	<0.0001	0.758	0.806	0.710	0.774 (0.650–0.871)	<0.0001
	LR	0.753	0.775	0.732	0.847 (0.777–0.902)	<0.0001	0.742	0.742	0.742	0.768 (0.643–0.866)	<0.0001

ACC, accuracy; SEN, sensitivity; SPE, specificity; AUC, the area under the curve.

Among the five ultrasound radiomics models, the DT classifier exhibited the best predictive performance, with an AUC value of 0.778 and an accuracy of 0.774 in the test set. The AUC values of the ultrasound radiomics models based on XGBoost, SVM, RF, and LR were 0.745, 0.768, 0.738, and 0.684, respectively, with corresponding accuracies of 0.710, 0.726, 0.742, and 0.661.

Among the five clinical models, the RF classifier performed best, with an AUC value of 0.761 and an accuracy of 0.710 in the test set. The AUC values of XGBoost, SVM, DT, and LR-based clinical models were 0.739, 0.688, 0.744, and 0.726, respectively, with corresponding accuracies of 0.661, 0.661, 0.726, and 0.661.

### 3.4 Predictive performance of combined models

The predictive performance of the combined models is shown in Table 2 and Figures 5C,F. The AUC values of XGBoost, SVM, RF, DT, and LR-based combined models in the test set were 0.846, 0.774, 0.853, 0.778, and 0.768, respectively. The XGBoost and RF models exhibited higher AUC values. However, the accuracy and sensitivity of the XGBoost model were 0.823 and 0.806, which were significantly superior to those of the RF model (0.774 and 0.710). Therefore, the XGBoost-based combined model performed best.

### 3.5 Comparison of performance of different models

**Figure 6:**
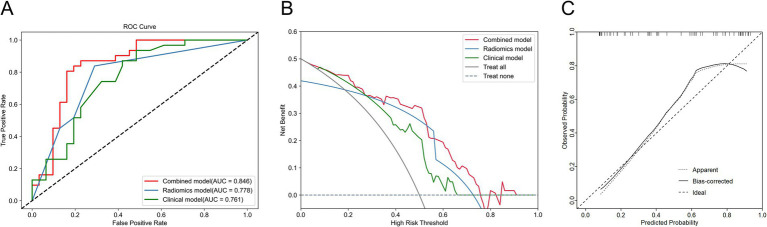
Comparison of the performance of three models in the test set. **(A)** ROC curve of three models. **(B)** Decision curve analysis (DCA) for three models. **(C)** Calibration curve of the combined model.

**Figure 7:**
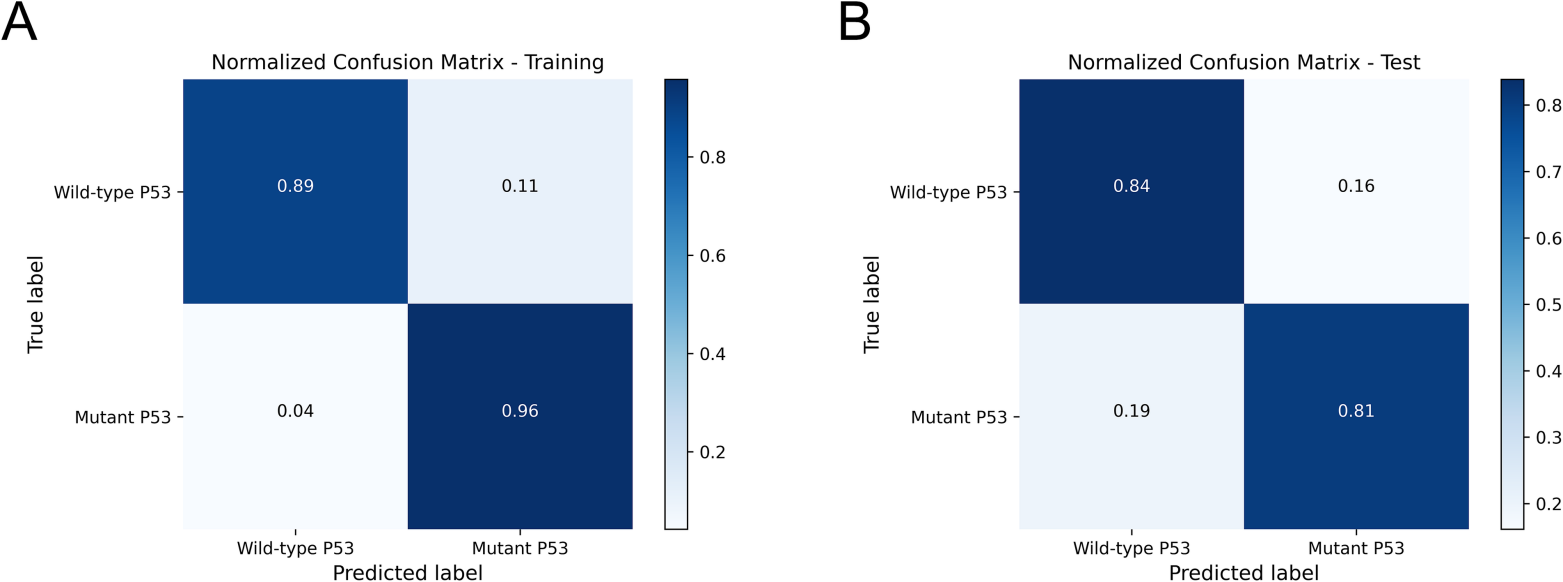
The confusion matrix of the combined model in the training set **(A)** and the test set **(B)**.

We compared and analyzed the performance of the optimal ultrasound radiomics model, the optimal clinical model, and the optimal combined model. As shown in Figure 6A, the combined model demonstrated optimal predictive performance among the three models, with a higher AUC value in the test set (0.846). DCA demonstrated that the combined model provided superior clinical net benefit, indicating its higher utility in clinical practice (Figure 6B). The calibration curve showed that the combined model had a satisfactory agreement between the predicted *TP53* status and the actual *TP53* status (Figure 6C). The confusion matrix showed that the combined model effectively distinguished both wild-type *TP53* and mutant *TP53*, without any class bias (Figure 7).

## 4 Discussion

*TP53* mutation is one of the most common gene mutations in HCC, which plays an important role in tumor development, metastasis, and the regulation of tumor microenvironment (TME) (5). In routine clinical practice, the determination of *TP53* gene status primarily relies on IHC of surgical resection specimens or biopsy tissues. While IHC is a reliable and standardized method for assessing *TP53* status, its invasive nature, delayed diagnosis, and high cost may limit the prevalence of its clinical detection. Therefore, there is a need for a noninvasive and cost-effective method for the preoperative assessment of *TP53* gene status. Kitao et al. found that vasodilatation in the arterial phase of dynamic CT and relatively low signal in the hepatobiliary phase of gadoxetic acid-enhanced MRI were independent predictors of *TP53*-mutated HCC (AUC: 0.770) (41). Wu et al. performed texture analysis on CT images of HCC patients and found that texture parameters had a predictive effect on *TP53* mutation (AUC: 0.621–0.792) (32). In the current study, the ultrasound radiomics model and the combined model achieved AUC of 0.778 and 0.846, respectively, demonstrating similar performance to CT and MRI models. The results indicated that ultrasound images contained significant information related to *TP53* mutation and possessed substantial potential for clinical application. Furthermore, compared with CT or MRI, ultrasound offers advantages such as real-time imaging, convenience, non-radiation, and lower cost, making it a more suitable imaging tool for large-scale screening and long-term monitoring.

Ultrasound radiomics technology extracts and analyzes quantitative features from medical images, thereby uncovering potential biological information and enabling a comprehensive evaluation of tumor heterogeneity. An ultrasound radiomics study conducted by Zhang et al. to predict the expression status of Ki-67 in HCC showed that the ultrasomics model (AUC: 0.861, accuracy: 0.674) outperformed the clinical model (AUC: 0.700, accuracy: 0.651) (42). This finding aligned with our study results. The superior diagnostic performance of radiomics arises from its ability to detect subtle phenotypic variations and spatial heterogeneity, thereby providing a more comprehensive diagnostic perspective compared to conventional clinical features. Wu et al. only analyzed the correlation between texture parameters of CT images and *TP53*-mutated HCC (32). However, other radiomics features, such as higher-order features, also play a significant role in the evaluation of HCC. In this study, we extracted and analyzed all types of ultrasound radiomics features. Finally, 24 most significant ultrasound radiomics features were selected, of which 21 were higher-order features and the remaining features were first-order features and texture features. First-order features reflect the distribution of voxel intensity values, such as mean, minimum, and skewness. Texture features, namely second-order features, describe the spatial relationships between voxels exhibiting similar gray values within ROI, which reflect tumor heterogeneity. Common texture features include GLCM, GLDM, and others (43). Higher-order features refer to radiomics features extracted from filter-processed images, capturing more complex details and more clearly reflecting subtle changes within the tumor (44). Among the 21 higher-order features extracted, 17 were wavelet features, while the rest were square root, logarithmic, and exponential features. Our results demonstrate that wavelet features are crucial for predicting *TP53* mutation status, consistent with previous studies (30). Wavelet features may capture complex information associated with the *TP53* mutation status in HCC. The wavelet filter decomposes the original image into high-frequency and low-frequency sub-images, allowing for multi-scale analysis through wavelet functions. Wavelet transform enables a deeper understanding of the spatial heterogeneity of tumors (45). Previous studies have also demonstrated that wavelet features are powerful tools for analyzing image information and hold significant value in radiomics research (35, 46). Furthermore, incorporating clinical features with ultrasound radiomics features improved predictive performance, highlighting the complementary role of clinical data in radiomics models. This aligned with previous studies, which have demonstrated that integrating multi-source information enhances the robustness and generalizability of predictive models (47–49).

The dominance of the XGBoost-based combined model may be attributed to its intrinsic compatibility with multi-source features. XGBoost, an advanced gradient boosting algorithm, offers notable advantages in scalability and training speed. It incorporates regularization terms and second-order Taylor expansion in the objective function, effectively controlling model complexity, mitigating overfitting, and enhancing generalizability and predictive accuracy (50). Consistent with our finding, previous radiomics studies have also reported superior performance of the XGBoost classifier compared to other classifiers, further supporting the reliability of our modeling approach (48, 49).

Our study presented a novel approach for the preoperative identification of high-risk HCC patients with *TP53* mutations. This study may be helpful in the following aspects. First, surgeons should consider a more aggressive surgical approach for HCC patients predicted to have *TP53* mutations, due to the heightened risk of recurrence and microvascular invasion (51). Second, oncologists may adjust treatment strategies, as *TP53* mutations are linked to resistance to certain systemic therapies, highlighting the potential need for alternative targeted treatments or combination therapies (52). Third, radiologists should conduct more rigorous imaging surveillance to detect early tumor progression or recurrence. Meanwhile, pathologists need to meticulously assess tumor characteristics in pathological specimens, combining the radiomics prediction, to provide more precise diagnostic information (53). Finally, patients predicted to have *TP53* mutations should adhere to a personalized treatment regimen and undergo intensive follow-up.

There were some limitations in this study. First, as a retrospective single-center study, it had a limited sample size and lacked external validation. In the future, we plan to conduct a large-scale, multi-center study to validate the generalizability of this model. Second, due to the retrospective nature, there were variations in ultrasound equipment and scanning parameters. Despite image preprocessing and ICC tests, potential confounding factors may still have influenced the results. Third, this study employed IHC to assess *TP53* gene status instead of gene sequencing technology. Although IHC is widely used in clinical practice due to its feasibility and cost-effectiveness, its ability to differentiate *TP53* gene status is limited. To enhance the accuracy and reliability of future research, gene sequencing technology will be incorporated to precisely determine *TP53* mutation status. Additionally, this study only used gray-scale ultrasound images in the current study and did not include contrast-enhanced ultrasound, elastography, CT, or other images. In the future, we plan to conduct a multi-modal radiomics study to enhance predictive performance and clinical applicability.

## 5 Conclusion

The machine learning-based ultrasound radiomics model was able to effectively predict the *TP53* mutation status in HCC. When combined with clinical information, the performance of the ultrasound radiomics model can be further enhanced. The XGBoost-based combined model exhibited the highest predictive performance, highlighting its potential as a powerful tool for *TP53* mutation prediction. While these findings require validation with larger sample sizes, ultrasound radiomics provides a non-invasive and efficient approach for detecting *TP53* gene mutation. This approach facilitates the preoperative screening of high-risk individuals for *TP53* mutation and aids in the development of personalized treatment strategies for HCC patients.

## Data Availability

The original contributions presented in the study are included in the article/Supplementary material, further inquiries can be directed to the corresponding author.

## Glossary

HCC: Hepatocellular carcinoma
ICC: Intraclass correlation coefficient
XGBoost: Extreme gradient boosting
SVM: Support vector machine
RF: Random forest
DT: Decision tree
LR: Logistic regression
AUC: Area under the curve
DCA: Decision curve analysis
TME: Tumor microenvironment
AFP: Alpha-fetoprotein
ALT: Alanine aminotransferase
AST: Aspartate aminotransferase
ALB: Albumin level
TBIL: Total bilirubin
PT: Prothrombin time
DICOM: Digital Imaging and Communications in Medicine
GLCM: Gray-level co-occurrence matrix
GLDM: Gray-level dependence matrix
GLRLM: Gray-level run length matrix
GLSZM: Gray-level size-zone matrix
NGTDM: Neighboring gray-tone-difference matrix
MIC: Maximal Information Coefficient
ROI: Region of interest
