# Supplementary material for: Machine learning-based ultrasound radiomics for predicting TP53 mutation status in hepatocellular carcinoma
Source: Front Med (Lausanne). 2025 Apr 28;12:1565618. doi: 10.3389/fmed.2025.1565618 (PMC12066593; doi:10.3389/fmed.2025.1565618)
Supplement: Supplementary file 1 [file Table_1.docx]

**Supplementary Table 1.** Baseline features of lesions in the training set and the test set.

| **Characteristic** | **Overall** | **Training set** | **Test set** | ***p*** |
| --- | --- | --- | --- | --- |
| Age (years) | 57.97±9.24 | 58.21±9.38 | 57.06±8.91 | 0.446 |
| Sex |  |  |  | 0.543 |
| Male | 146(80.2) | 102(79.1) | 44(83.0) |  |
| Female | 36(19.8) | 27(20.9) | 9(17.0) |  |
| HBsAg |  |  |  | 0.251 |
| Negative | 41(22.5) | 32(24.8) | 9(17.0) |  |
| Positive | 141(77.5) | 97(75.2) | 44(83.0) |  |
| Child-Pugh class |  |  |  | 0.766 |
| A | 160(87.9) | 114(88.4) | 46(86.8) |  |
| B-C | 22(12.1) | 15(11.6) | 7(13.2) |  |
| Liver cirrhosis |  |  |  | 0.523 |
| No | 23(12.6) | 15(11.6) | 8(15.1) |  |
| Yes | 159(87.4) | 114(88.4) | 45(84.9) |  |
| Portal hypertension |  |  |  | 0.016 |
| No | 124(68.1) | 81(62.8) | 43(81.1) |  |
| Yes | 58(31.9) | 48(37.2) | 10(18.9) |  |
| AFP (ng/mL) |  |  |  | 0.995 |
| <400 | 127(69.8) | 90(69.8) | 37(69.8) |  |
| >400 | 55(30.2) | 39(30.2) | 16(30.2) |  |
| ALT (U/L) | 28.10(22.2,49.5) | 27.70(22.4,48.3) | 30.30(22.2,50.1) | 0.912 |
| AST (U/L) | 33.50(23.4,49.5) | 33.70(23.7,50.4) | 32.10(22.9,46.8) | 0.509 |
| ALB (g/L) | 39.60(35.6,43.3) | 39.30(36.0,43.3) | 40.10(34.7,43.7) | 0.978 |
| TBIL (μmol/L) | 13.10(9.4,19.3) | 13.60(10.3,19.7) | 11.40(8.7,15.5) | 0.039 |
| GGT (U/L) | 59.10(30.0,127.4) | 57.80(29.9,132.3) | 61.80(29.6,117.8) | 0.872 |
| PT (s) | 12.50(11.9,13.3) | 12.50(11.9,13.4) | 12.40(11.9,13.2) | 0.475 |
| Splenomegaly |  |  |  | 0.371 |
| No | 104(57.1) | 71(55.0) | 33(62.3) |  |
| Yes | 78(42.9) | 58(45.0) | 20(37.7) |  |
| Tumor diameter (mm) | 34.00(22.0,54.0) | 34.00(21.5,57.0) | 33.00(22.5,51.0) | 0.671 |
| Echo signal |  |  |  | 0.105 |
| Low | 91(50) | 71(55.0) | 20(37.7) |  |
| Equal | 33(18.1) | 21(16.3) | 12(22.6) |  |
| High | 58(31.9) | 37(28.7) | 21(39.6) |  |
| Margin |  |  |  | 0.266 |
| Clear | 111(61.0) | 82(63.6) | 29(54.7) |  |
| Obscure | 71(39.0) | 47(36.4) | 24(45.3) |  |
| Shape |  |  |  | 0.683 |
| Regular | 85(46.7) | 59(45.7) | 26(49.1) |  |
| Irregular | 97(53.3) | 70(54.3) | 27(50.9) |  |
| Doppler flow signal |  |  |  | 0.571 |
| No | 119(65.4) | 86(66.7) | 33(62.3) |  |
| Yes | 63(34.6) | 43(33.3) | 20(37.7) |  |
| Tumor location |  |  |  | 0.746 |
| Left lobe | 30(16.5) | 22(17.1) | 8(15.1) |  |
| Right lobe | 152(83.5) | 107(82.9) | 45(84.9) |  |

HBsAg, hepatitis B surface antigen; AFP, alpha-fetoprotein; ALT, alanine aminotransferase; AST, aspartate aminotransferase; ALB, albumin; TBIL, total bilirubin; GGT, Gamma-glutamyltransferase; PT, prothrombin time; *p,* difference of clinical features between the training set and the test set.
