# Supplementary material for: Machine learning-based ultrasound radiomics for predicting TP53 mutation status in hepatocellular carcinoma
Source: Front Med (Lausanne). 2025 Apr 28;12:1565618. doi: 10.3389/fmed.2025.1565618 (PMC12066593; doi:10.3389/fmed.2025.1565618)
Supplement: Supplementary file 2 [file Table_2.DOCX]

**Supplementary Table 2.** Weight coefficients of 24 the radiomics features.

| **Feature name** | **Weight coefficients** |
| --- | --- |
| original_firstorder_Minimum | 0.07305053 |
| original_glcm_Correlation | 0.04813728 |
| original_glrlm_RunLengthNonUniformity | 0.02211328 |
| wavelet-LLH_firstorder_Kurtosis | 0.03254440 |
| wavelet-LLH_firstorder_RobustMeanAbsoluteDeviation | 0.02213150 |
| wavelet-LLH_glszm_ZonePercentage | 0.03668725 |
| wavelet-LHL_firstorder_10Percentile | 0.03657434 |
| wavelet-LHL_firstorder_Median | 0.03244198 |
| wavelet-LHL_firstorder_RootMeanSquared | 0.05168303 |
| wavelet-LHH_glszm_SizeZoneNonUniformity | 0.05849640 |
| wavelet-HLL_glrlm_LongRunLowGrayLevelEmphasis | 0.02921389 |
| wavelet-HLL_gldm_DependenceVariance | 0.04326160 |
| wavelet-HHL_glcm_SumSquares | 0.03088548 |
| wavelet-HHL_glrlm_RunEntropy | 0.03415875 |
| wavelet-HHL_gldm_LargeDependenceLowGrayLevelEmphasis | 0.04766097 |
| wavelet-HHH_firstorder_Kurtosis | 0.03933357 |
| wavelet-HHH_firstorder_Range | 0.03575652 |
| wavelet-HHH_glcm_ClusterShade | 0.04185334 |
| wavelet-LLL_glcm_Correlation | 0.05755718 |
| wavelet-LLL_glszm_SizeZoneNonUniformity | 0.04735401 |
| squareroot_firstorder_Skewness | 0.03807553 |
| logarithm_firstorder_InterquartileRange | 0.06593379 |
| logarithm_firstorder_MeanAbsoluteDeviation | 0.03494851 |
| exponential_firstorder_Mean | 0.04014682 |
