# Supplementary material for: Machine learning-based ultrasound radiomics for predicting TP53 mutation status in hepatocellular carcinoma
Source: Front Med (Lausanne). 2025 Apr 28;12:1565618. doi: 10.3389/fmed.2025.1565618 (PMC12066593; doi:10.3389/fmed.2025.1565618)
Supplement: Supplementary file 3 [file Table_3.DOCX]

Supplementary Material 1

**Preparation of p53 immunohistochemical sections**

All hepatocellular carcinoma (HCC) specimens were fixed in 10% formalin, dehydrated conventionally, paraffin-embedded, and cut into 4 μm thick sections. Immunohistochemical staining was performed with an anti-p53 monoclonal antibody (Maixin, Cat. MAB-0674) .

**Detection methods of laboratory indicators**

The detection of laboratory indicators was performed using various automated analyzers.

Hepatitis B surface antigen (HBsAg) and alpha-fetoprotein (AFP) levels were determined with an automatic chemiluminescence immunoassay analyzer (AutoLumo A6200, Autobio, Zhengzhou, China), using the corresponding reagents for HBsAg (Autobio, Cat. CMC0102) and AFP (Autobio, Cat. CMB0102).

Liver function markers, including aspartate aminotransferase (AST), alanine aminotransferase (ALT), total bilirubin (TBIL), albumin level (ALB), and gamma-glutamyltransferase (GGT) were measured using an automatic biochemical analyzer (Architect c16000, Abbott, Chicago, Illinois, USA), with the following reagents: AST (Abbott, Cat. 7D81-21), ALT (Abbott, Cat. 7D56-21), TBIL (Abbott, Cat. 6L45-21), ALB (Abbott, Cat. 7D53-22), and GGT (Abbott, Cat. 7D65-21).

Prothrombin time (PT) were assessed with a coagulation detection analyzer (ACL TOP 750, Werfen, Barcelona, Spain), using the PT reagent (Werfen, Cat. 0020301300).
