# Supplementary material for: Machine learning-based ultrasound radiomics for predicting TP53 mutation status in hepatocellular carcinoma
Source: Front Med (Lausanne). 2025 Apr 28;12:1565618. doi: 10.3389/fmed.2025.1565618 (PMC12066593; doi:10.3389/fmed.2025.1565618)
Supplement: Supplementary file 4 [file Table_4.DOCX]

Supplementary Material 2

**Parameter selection of the models**

Parameter selection plays a crucial role in optimizing the performance of machine learning models. We used grid search and random search to select the best parameters for multiple machine learning models. The best parameters for each model are as follows.

**Best parameters in the clinical model**

Best parameters for XGBoost: {‘verbosity’: 0, ‘n_estimators’: 3, ‘objective’: 'binary: logistic', ‘learning_rate’: 0.1, ‘gamma’: 0, ‘max_depth’: 4, ‘subsample’: 1, ‘colsample_bytree’: 1, ‘colsample_bylevel’: 1, ‘scale_pos_weight’: 1, ‘reg_lambda’: 1, ‘reg_alpha’: 0, ‘booster’: 'gbtree', ‘n_jobs’: -1, ‘eval_metric’: ['logloss','auc','error']}

Best parameters for SVM: {‘kernel’: "rbf", ‘C’: 1.4174741629268048, ‘gamma’: 0.07564633275546291, ‘cache_size’: 5000, ‘class_weight’: {1: 1}}

Best parameters for RF: {‘n_estimators’: 14, ‘max_depth’: 2, ‘random_state’: 6}

Best parameters for DT: {‘criterion’: "gini", ‘splitter’: "best", ‘max_depth’: 5, ‘random_state’: 0, ‘class_weight’: None}

Best parameters for LR: {‘penalty’: 'l2', ‘C’: 0.13219411484660287, ‘solver’: 'liblinear', ‘max_iter’: 100000000}

**Best parameters in the ultrasound radiomics model**

Best parameters for XGBoost: {‘verbosity’: 0, ‘n_estimators’: 35, ‘objective’: 'binary: logistic', ‘learning_rate’: 0.1, ‘gamma’: 0, ‘max_depth’: 2, ‘subsample’: 1, ‘colsample_bytree’: 1, ‘colsample_bylevel’: 1, ‘scale_pos_weight’: 1, ‘reg_lambda’: 1, ‘reg_alpha’: 0, ‘booster’: 'gbtree', ‘n_jobs’: -1, ‘eval_metric’: ['logloss','auc','error']}

Best parameters for SVM: {‘kernel’: "rbf", ‘C’: 11.497569953977356, ‘gamma’: 0.010722672220103232, ‘cache_size’: 5000, ‘class_weight’: {1: 1}}

Best parameters for RF: {‘n_estimators’: 37, ‘max_depth’: 2, ‘random_state’: 0}

Best parameters for DT: {‘criterion’: "gini", ‘splitter’: "best", ‘max_depth’: 7, ‘random_state’: 9, ‘max_features’: 'sqrt', ‘class_weight’: {0: 1,1: 1.2}}

Best parameters for LR: {‘penalty’: 'l2', ‘C’: 3.7649358067924674, ‘solver’: 'saga', ‘multi_class’: 'multinomial', ‘max_iter’: 100000000}

**Best parameters in the combined model**

Best parameters for XGBoost: {‘verbosity’: 0, ‘n_estimators’: 92, ‘objective’: 'binary: logistic', ‘learning_rate’: 0.2, ‘gamma’: 0, ‘max_depth’: 1, ‘subsample’: 0.8, ‘colsample_bytree’: 1, ‘colsample_bylevel’: 1, ‘scale_pos_weight’: 1, ‘reg_lambda’: 1, ‘reg_alpha’: 0, ‘booster’: 'gbtree', ‘n_jobs’: -1, ‘eval_metric’: ['logloss','auc','error']}

Best parameters for SVM: {‘kernel’: "rbf", ‘C’: 15.199110829529332, ‘gamma’: 0.005336699231206312, ‘cache_size’: 5000, ‘class_weight’: {1: 1}}

Best parameters for RF: {‘n_estimators’: 27, ‘max_depth’: 2, ‘random_state’: 0}

Best parameters for DT: {‘criterion’: "gini", ‘splitter’: "best", ‘max_depth’: 6, ‘random_state’: 0, ‘class_weight’: None}

Best parameters for LR: {‘penalty’: 'l2', ‘C’: 3.4304692863149193, ‘solver’: 'saga', ‘max_iter’: 100000000}
